# Development and validation of a novel disulfidptosis-related lncRNAs signature in patients with HPV-negative oral squamous cell carcinoma

**DOI:** 10.1038/s41598-024-65194-y

**Published:** 2024-06-23

**Authors:** Fan Yang, Xinyu Niu, Mingzhu Zhou, Wei Li

**Affiliations:** 1 Department of Geriatrics, Union Hospital, Tongji Medical College, Huazhong University of Science and Technology, Wuhan, 430022 China; 2 Department of Otorhinolaryngology, Union Hospital, Tongji Medical College, Huazhong University of Science and Technology, Wuhan, 430022 China

**Keywords:** Cancer, Long non-coding RNAs, Disulfidptosis, Medical research, Cancer, Computational biology and bioinformatics, Oncology

## Abstract

Disulfidptosis is a recently identified mode of regulated cell death. Regulating disulfidptosis in carcinoma is a promising therapeutic approach. Long non-coding RNAs (lncRNAs) have been reported to be related to the occurrence and development of many cancers. Disulfidptosis-related lncRNAs (DRLs) in HPV-negative oral squamous cell carcinoma (OSCC) have not been studied. Based on The Cancer Genome Atlas (TCGA) database, least absolute shrinkage selection operator (LASSO) analysis and Cox regression analysis were used to identify overall survival related DRLs and construct the signature. Kaplan–Meier, time-dependent receiver operating characteristics (ROC) and principal component analyses (PCA) were explored to demonstrate the prediction potential of the signature. Subgroup analysis stratified by different clinicopathological characteristics were conducted. Nomogram was established by DRLs signature and independent clinicopathological characteristics. The calibration plots were performed to reveal the accuracy of nomogram. Immune cell subset infiltration, immunotherapy response, drug sensitivity analysis, and tumor mutation burden (TMB) were conducted. Underlying functions and pathways were explored by Gene Set Enrichment Analysis (GSEA) analysis. Previous lncRNA signatures of OSCC were retrieved from PubMed for further validation. Gene expression omnibus (GEO) datasets (GSE41613 and GSE85446) were merged as an external validation for DRLs signature. Consensus clustering analysis of DRLs signature and experimental validation of DRLs were also explored. This research sheds light on the robust performance of DRLs signature in survival prediction, immune cell infiltration, immune escape, and immunotherapy of HPV-negative OSCC.

## Introduction

Oral squamous cell carcinoma (OSCC) is a sort of prevalent head and neck malignancy, most derived from the oral cavity, including the regions of lips, tongue, palate, cheek mucosa, gum, the bottom and vestibule of the mouth, and retromolar^1^. Global Cancer Observatory (GCO) reported 377,713 OSCC new cases with 177,757 deaths worldwide in 2020^2^. Although human papillomavirus (HPV) infection attributed to the overwhelming incidence of OSCC over the past decades^3^. HPV-negative disease still accounts for the majority of OSCC and improving the outcome of this subset is still an area of unmet need. HPV-negative OSCC have significantly poorer prognosis than HPV-positive OSCC^4^. The mortality rate of HPV-negative OSCC is still rising, with survival rate of 5-years less than 40% compared to 60% survival rate of HPV-positive OSCC^5^. The recurrence rate of majority patients with HPV-negative OSCC is up to 76% under multidisciplinary treatment^6^. To improve HPV-negative OSCC survival, it is critical to explore novel molecular events and establish a solid signature.

Long non-coding RNAs (LncRNAs) are known since the 1950s and defined as RNA transcripts > 200 nucleotides without encoding protein functions^7,8^. In the past few years, a series of advent of high-throughput and sensitive technologies have revealed that lncRNAs can act as co-factors of transcription factors and enzymes that regulating gene expression in the nucleus and cytoplasm. The mutations and dysregulated expression of lncRNAs can enhance or inhibit cancer functions^9^. Increasing evidence has revealed that lncRNAs fulfill the regulatory functions that influence cell biological behaviors. For instance, lncRNA AC007271.3 is reported to promote cell proliferation, migration, and invasion or inhibit cell apoptosis of OSCC via the Wnt/β-catenin signaling pathway^10^. LncRNA GACAT1 is involved in the apoptosis and autophagy of OSCC by targeting miRNA-149^11^.

A latest study published in the journal *Nature Cell Biology*, Liu et al.^12^ uncovered a novel form of regulated cell death (RCD) recognized as disulfidptosis. Under glucose starvation conditions, solute carrier family 7member 11 (SLC7A11) accelerates nicotinamide adenine dinucleotide phosphate (NADPH) depletion in the cytoplasm. With accumulation of intracellular disulfide-like molecules such as cysteine, the disulfide bond content in the actin cytoskeleton consequential upregulate, causing collapse of the actin filaments to contract and the cytoskeletal structure, resulting disulfide stress and cell death^13^. In recent years, the discovery of disulfidptosis and its potential pathophysiological mechanism have provided new potential targets for cancer treatment. An increasing number of studies have shown that the SLC7A11 plays a crucial role in tumorigenesis and progression^14^. The anticancer effects of glucose transporter (GLUT) inhibitor monotherapy, which is considerably linked with the high expression level of SLC7A11 are explored in preclinical models and expected to be used in the clinic^15^. Disulfidptosis is expected to provide novel insights in cancer treatment.

The present study intended to identify disulfidptosis-related lncRNAs (DRLs) and develop a risk stratification signature for HPV-negative OSCC patients. This work could provide valuable insights for potential chemotherapeutic drugs and optimize the treatment for patients with HPV-negative OSCC.

## Materials and methods

**Figure 1:**
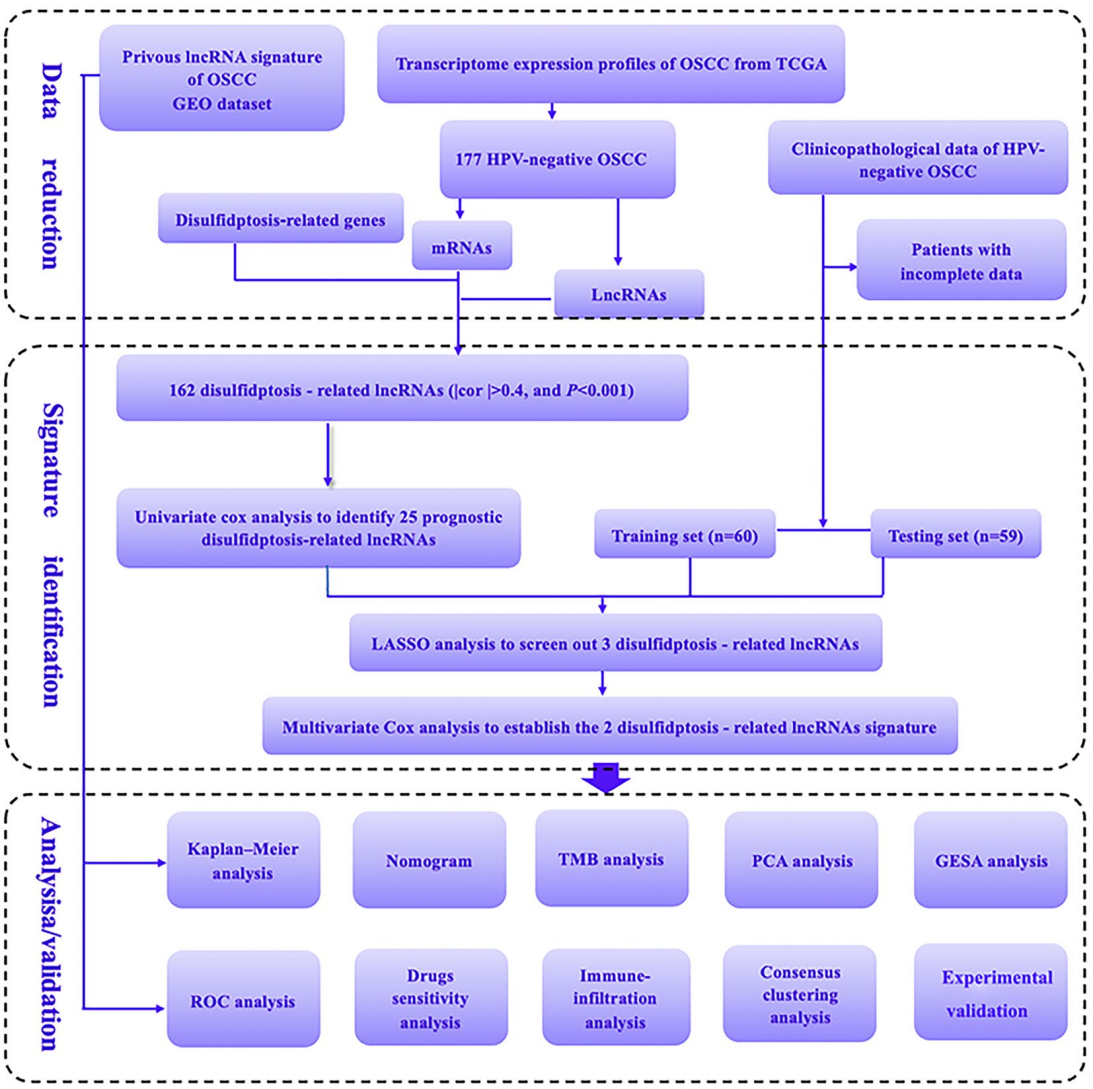
The workflow chart of the study.

The workflow chart of this study is depicted in Fig. 1.

### Data processing

Transcriptome expression data, clinicopathological characteristics, and somatic mutation data were downloaded from the The Cancer Genome Atlas (TCGA) database (https://tcga-data.nci.nih.gov/tcga/). The inclusion criteria of TCGA database were as follows: (1) Histologically verified as OSCC primary tumor; (2) Patients with HPV-supporting reads ≤ 100 were defined as HPV negative; (3) RNA expression profiles with missing values ≤ 30% of the total samples were retained. The exclusion criteria of TCGA database were as follows: (1) Samples with no data on survival status or survival time were excluded, the definition of survival time referred to Liu et al.^16^; (2) Patients without HPV status were excluded; (3) Patients with histories of malignancies and/or adjuvant therapies were also excluded. According to the inclusion and exclusion criteria, 177 HPV-negative OSCC from TCGA database were screened out for analysis. TCGA datasets were used for identification, while gene expression omnibus (GEO) datasets were chosen for external validation. Expression profiles and clinicopathological characteristics of HPV-negative OSCC were selected from the GEO database (https://www.ncbi.nlm.nih.gov/geo/). 97 HPV-negative OSCC from GSE41613 (GPL570 platform) and 66 HPV-negative OSCC from GSE85446 (GPL6480 platform) were merged as an external validation. The lncRNAs annotations were obtained from the website of GENCODE (https://www.gencodegenes.org/).Disulfidptosis-related genes(GYS1,NDUFS1,OXSM,LRPPRC,NDUFA11,NUBPL,NCKAP,RPN1,SLC3A2, and SLC7A11)were manually compiled from the previous literature^12^.

### Identification of disulfidptosis-related lncRNAs (DRLs) signature

Pearson correlation analysis was performed by the “limma” R package to evaluate the relationship between disulfidptosis-related genes and DRLs^17^. Using the “ggplot2,” “ggalluvial,” and “dply” R packages, the result of pearson correlation analysis was visualized as sankey diagrams based on the |Cor|> 0.4 and *P* < 0.001 criteria. Univariate COX regression analysis was performed using the “survival” R package to screen survival related DRLs. Using the “caret” R package, patients in the TCGA dataset were randomly split into training set and testing set. For the training cohort, LASSO analysis was conducted to refine survival related DRLs using the “glmnet” R package. Through 1000-fold cross-validation with a *P*-value of 0.05 of penalty parameter, the LASSO-related DRLs were identified. Subsequently, multivariate COX regression analysis was employed to determine the final DRLs for signature construction. The algorithm of the disulfidptosis-related riskscore was calculated as follows: $Risk score =\sum_{\text{coef}=\text{i}}^{2}exp\left(i\right) \times Coef(i)$, where coefi represents the coefficients and expi represents the expression level of each DRLs.

### Validation of DRLs signature

The risk score of each patient with HPV-negative OSCC can be calculated by DRLs signature. Based on the median riskscore of the training cohort, all TCGA patients were stepwise categorized into the high-risk group (≥ median) or low-risk group (< median). Using the “survminer” and “survival” R packages, Kaplan–Meier curves with the log-rank test were conducted to depict the overall survival (OS) in each of the three datasets: training set, testing set, and all TCGA set. Progression-free survival (PFS) was also performed among the three datasets: training set, testing set, and all TCGA set. To confirm the distribution of risk values among patients in high- and low-risk group and to presume the risk of death, scatter plots, risk curves, and heatmaps were conducted using “pheatmap” R package. Using the “time ROC” R package, the time-dependent receiver operating characteristic (ROC) curves and the area under the curve (AUC) were used to assess the accuracy and prognostic predictive value of the DRLs signature compared with other clinicopathological characteristics. Kaplan–Meier survival curves were further used to compare OS within different clinicopathological characteristics (age, gender, stage, and grade) subgroups.

### Construction and verification of nomogram

The independent of clinicopathological characteristics were validated by univariate and multivariate COX regression analyses. Using the “regplot” R package, prognostic clinicopathological characteristics (age, grade, stage, gender) of patients in TCGA and DRLs signature were integrated to construct the nomogram, which served as a prognostic predictive tool on 1-, 3- and 5-years OS. Using “rms” R package, the calibration curves were performed to assess the predict performance of nomogram. The diagonal of the calibration curves indicated the consistency of nomogram’s OS and observed OS^18^.

### Principal component analysis (PCA) and gene set enrichment analysis (GSEA) analysis

Using “scatterplot3D” R package, the risk definition and spatial distribution of patients were analyzed by the principal component analysis (PCA)^19^. Gene set enrichment analysis (GSEA) software (https://www.gsea-msigdb.org/gsea/datasets.jsp) was employed to scrutinize the enriched gene functions and potential pathway analysis between high- and low-risk groups using the “h.all.v2023.1.Hs.symbols” and “c2.cp.kegg.v2023.1.Hs.symbols.gmt”^20,21^. The *P* < 0.05 and the false discovery rate (FDR) < 0.05 were considered significantly enriched.

### Immune infiltration analysis and immunotherapy response

Seven immune infiltration assessment algorithms (TIMER, CIBERSORT, QUANTISEQ, MCPCOUNTER, XCELL, and EPIC) were used to assess immune cell infiltration between high- and low-risk group based on DRLs signature^22^. Immune infiltration levels and immune checkpoint gene expression levels between high- and low-risk group were shown in box line plots. Using the “gsva” R package, single-sample gene set enrichment analysis (ssGSEA) was performed to quantify the immune-related function between the two risk groups. Tumor Immune Dysfunction and Exclusion (TIDE) platform (http://tide.dfci.harvard.edu/)^23^ was exploited to forecast the immunological evasion capacity of HPV-negative OSCC patient.

### Tumor mutation analysis

Tumor mutational burden (TMB) was defined as the number of mutated bases per one million bases. TMB of each TCGA patients were calculated and the median of the TMB value was set as cut-off point to divide patients into high-TMB or low-TMB group. Previous research has approved that the higher of TMB, the more benefit of patients from immunotherapy^24^. TMB has been considered as a predictive biomarker for immune checkpoint inhibitors^25^. To determine differences in gene alteration between the two risk groups, 15 genes with the highest tumor mutation frequency (TMF) were mapped using the " Maftools " R package. Comparative analysis and survival analysis were further employed between the different risk groups with TMB (*P* < 0.05). Using "survival" and "survminer" R packages, Kaplan–Meier curves were employed to clarify the effect of risk score and TMB on OS of patients with HPV-negative OSCC.

### Drug sensitivity analysis

The “oncoPredict” and “parallel” R packages were used for predicting drug sensitivity in HPV-negative OSCC patients. The anticancer drug dataset were obtained from the Genomics of Drug Sensitivity in Cancer database (https://osf.io/c6tfx/)^26^. CalcPhenotype function were employed to obtain drug sensitivity scores of each patient and the difference between the high- and low-risk groups were compared by the Wilcoxon signed-rank test^27^.

### Consensus clustering analysis of DRLs

Based on the DRLs signature, unsupervised consensus clustering analysis are estimated using the “ConsensusCluster” R package^28^. Using the “CIBERSORT” R package, the infiltration levels of immune cell subsets were estimated and visualized in boxplot and heatmap^29^. The attribute changes between disulfidptosis-related clusters and DRLs signature were visualized in alluvial diagram. The immune infiltration, immune checkpoints, and drug sensitivity analysis in the two different clusters were also evaluated.

### External validation

Previously established lncRNA signatures of OSCC from PubMed were retrieved and obtained the relevant lncRNAs for external validation of our signature. The GEO datasets (GSE41613 and GSE85446) were merged for further validation. Kaplan–Meier and ROC curves were used to compare the DRLs signature with other signatures and validate the accuracy of DRLs signature in GEO database.

### Validation of the expression level of DRLs by qRT-PCR

HPV-negative OSCC specimens and adjacent normal specimens were collected from five patients who suffered from operation at Wuhan Union Hospital. Diagnoses of HPV-negative primary OSCC were confirmed by two pathologists. This study was approved by the Huazhong University of Science and Technology Ethics Committee. All patients were signed informed consent forms prior to surgery, aligning with The Declaration of Helsinki guidelines for ethical considerations and patient safety. Collected specimens were immediately frozen in liquid nitrogen and stored at − 80 °C refrigerator. Total RNA was isolated using Trizol (Invitrogen, China). The reverse transcription and SYBR green were performed by the Prime Script RTase (Takara, China). Relative expression levels of lncRNAs were normalized using β-actin and repeated independently more than three times. The primer sequences applied in this study are depicted as follows:

β-actin, Forward-5'-GTTGTCGACGACGAGCG-3', Reverse-5'-GCACAGAGCCTCGCCTT-3'. AC104794.3, Forward-5'-GCGGCCCTAGATGTAACGAT-3', Reverse-5'-CCAATTGATTGGCGCTGTCC-3'. AL109936.2, Forward-5'-AGGTCCCATTCTGCCCTACT-3', Reverse-5'-AGTGCTCCAAACTGTGGCTT-3'.

### Cells culture

Normal human oral keratinocytes (NOK) and OSCC cell lines (SCC-9, SCC-15, and SCC-25) were obtained from the Institute of Biochemistry and Cell Biology of the Chinese Academy of Sciences (Shanghai, China). NOK, SCC-9, SCC-15, and SCC-25 cell lines were all cultured in Dulbecco's modified Eagle medium (DMEM, Gibco) supplemented with 10% fetal bovine serum (FBS, Gibco). All cells were incubated at 37 °C and 5% CO_2_.

### Cell transfection

The design and synthesis of lncRNA AC104794.3 overexpression plasmid (AC104794.3-OE) and its corresponding vector, were carried out by Sangon Biotech (Shanghai, China). Transfection was performed using Lipofectamine 3000 (Invitrogen) into SCC-9 cells (2.5 ug AC104794.3-OE plasmid and 5ul Lipofectamine 3000 in each well). After 48 h, the cells were collected for the subsequent experiments.

### Cell viability

SCC-9 cells were seeded into 96-well plates, with 100 μL DMEM culture medium (3 × 10^3^ cells/well). Cell viability was detected using the Cell Counting Kit-8 (Beyotime, Shanghai, China). After AC104794.3-OE transfection of 0 h, 24 h, 48 h, and 72 h, CCK-8 reagent (10μL per well) was added in each plate. The absorbance at a wavelength of 450 nm was measured following a two-hours incubation period (Bio-Rad).

### Cell proliferation

EdU incorporation assay kit (Beyotime, Shanghai, China) was applied to evaluate the rate of cell proliferation. SCC-9 cells were initially transfected by AC104794.3-OE and vector with 48 h and then incubated with EdU working solution for 2 h. Subsequently, cells were fixed with 4% paraformaldehyde and incubated for 30 min with Azide 555 and 0.5% Triton X100. Finally, Hoechst 33342 were used to stain the cell nucleuses. The percentage of EDU‐positive cells was calculated to assess the rate of proliferation.

### Cell migration

Cell migration was evaluated by the wound healing assay. SCC-9 cells were initially transfected by AC104794.3-OE and vector with 48 h and then seeded into 6-well plates. When cells grew to confluence at approximately 80%, 200 μL sterile pipette tip was used to create a wound. The wound-healing area were measured at the 0 h and after 24 h to assess cell migration.

### Statistical analysis

All bioinformatic analyses were performed using the R software (version 4.0.5). Student’s t test was conducted for two-group comparison. ANOVA was used to analyze potential differences for paired data with continuous variables. All experiments were repeated at least three times. *P* values < 0.05 were considered statistically significant.

## Results

### Identification of DRLs signature

**Figure 2:**
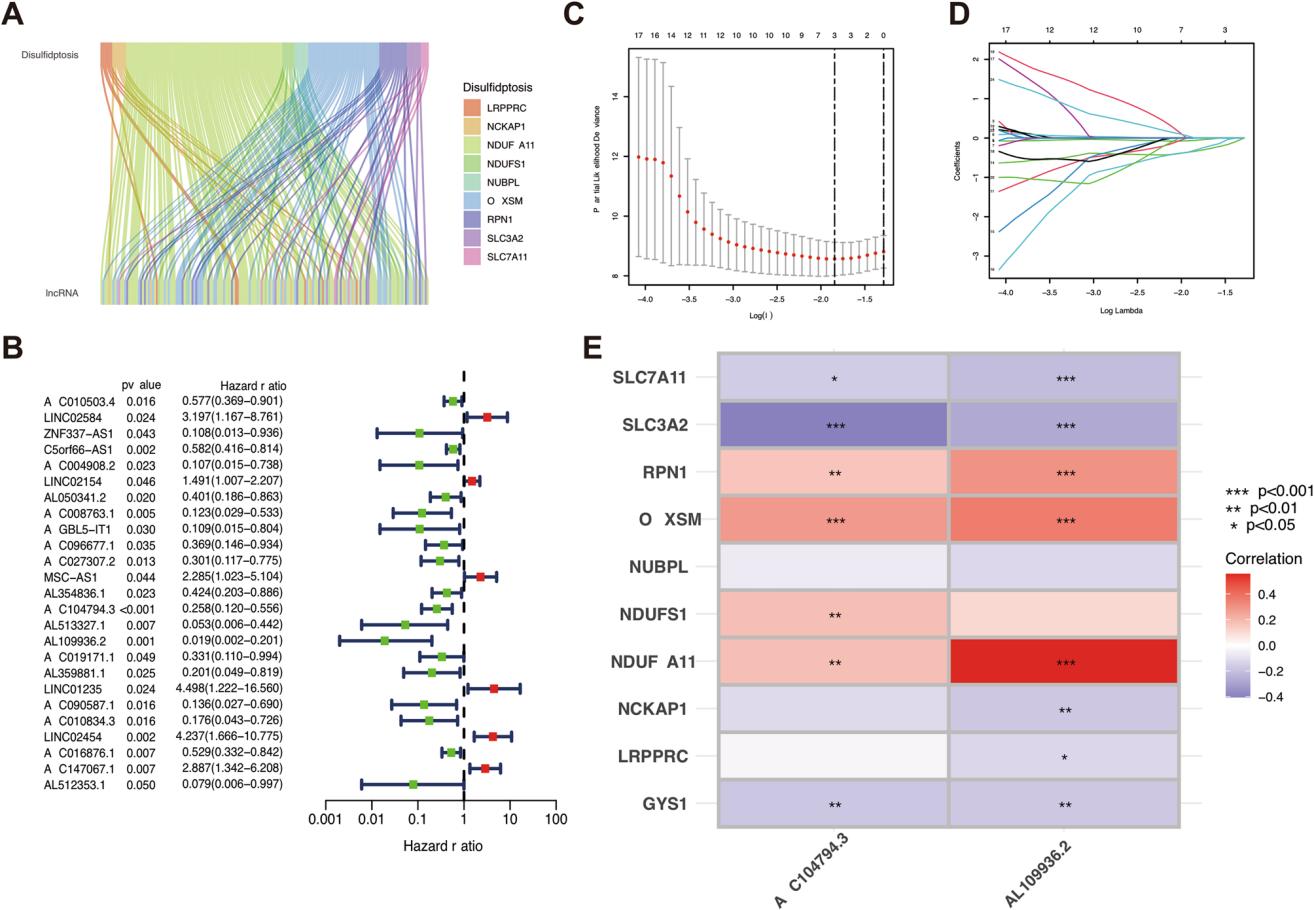
Identification of the disulfidptosis-related LncRNAs (DRLs) signature. (**A**) Sankey diagram of disulfidptosis-related genes and DRLs. (**B**) Forest plots of 25 DRLs selected by univariate COX regression analysis. (**C**) The LASSO coefficient profile of DRLs. (**D**) The tenfold cross-validation for variable selection in the LASSO model. (**E**) Heatmap for the correlations between DRLs and disulfidptosis-related genes. *P < 0.05, **P < 0.01, and ***P < 0.001.

Spearman correlation analysis identified 162 DRLs in the sankey diagrams (Fig. 2A). Univariate COX regression analysis was conducted to screen the relevance between DRLs and OS. Among the 25 differentially expressed DRLs, 6 DRLs were determined as a risk factor and the remaining 19 DRLs were identified as protective factors (Fig. 2B). Expression data and clinical data of HPV-negative OSCC were integrated and then patients were divided into training set (n = 60) and testing set (n = 59). LASSO regression analysis was stepwise applied to the training set. Three candidate DRLs of LASSO regression analysis were further screen by multivariate Cox regression analysis (Fig. 2C,D). Ultimately, two DRLs (AC104794.3 and AL109936.2) were identified to construct the risk signature. The risk score of each patient with HPV-negative OSCC were calculated based on the following formula: Risk Score = AC104794.3*(− 0.782850085135135) + AL109936.2*(− 2.89619560440594). Correlation between the DRLs signature and disulfidptosis-related genes were visualized in a heatmap, indicating a positive relationship of RPN1, OXSM, and NDUFA11 and a negative relationship of SLC7A11, GYS1 and SLC3A2 (Fig. 2E).

### Characteristics of DRLs signature

**Figure 3:**
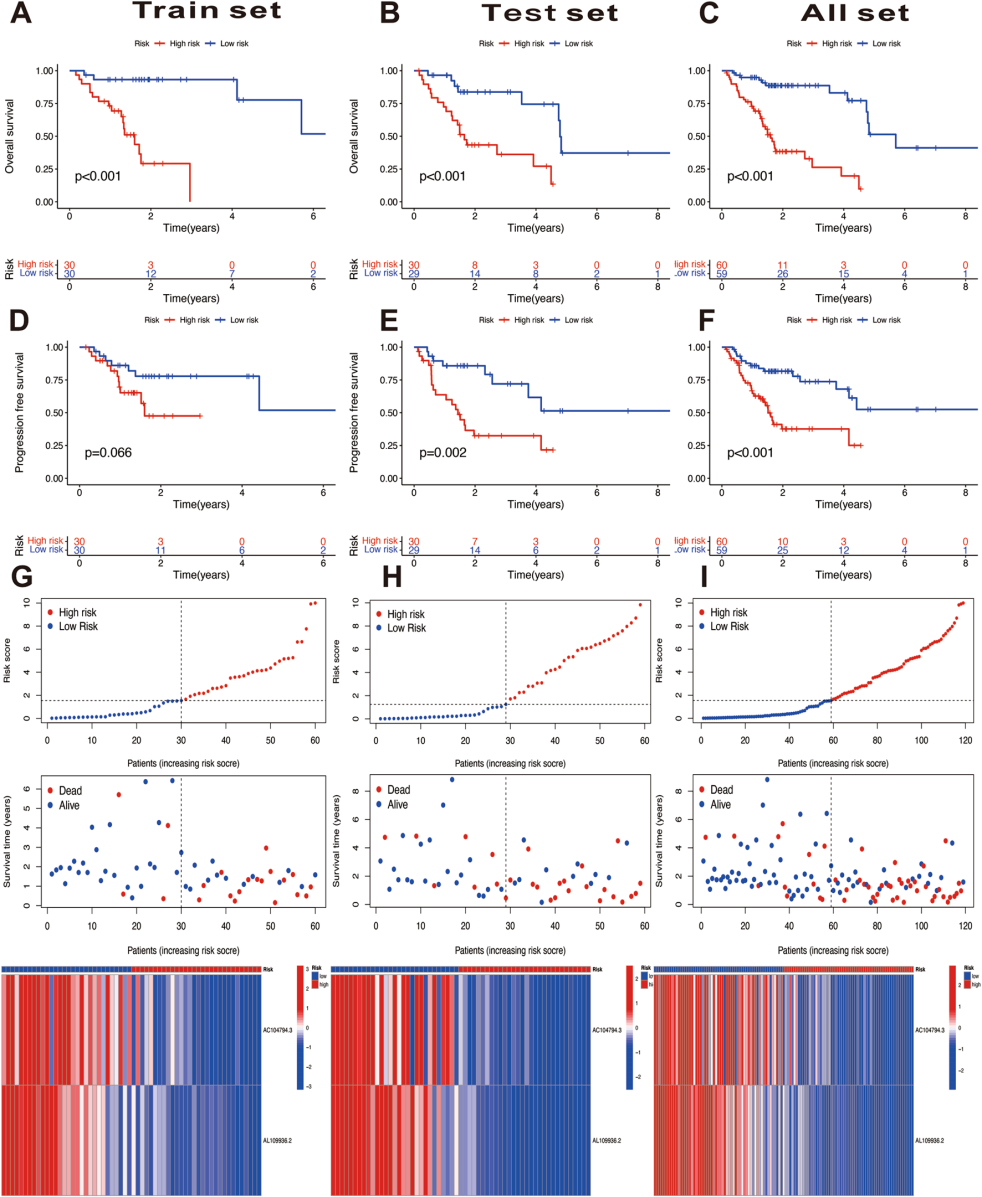
Survival analyses of disulfidptosis-related LncRNAs (DRLs) signature. (**A**–**C**) Kaplan–Meier curves of overall survival between the high- and low-risk group in the training set, test set, and all TCGA set. (**D**–**F**) Kaplan–Meier curves of progression free survival between the high-risk and low-risk group in the training set, test set, and all TCGA set. (**G**–**I**) The DRLs signature-based risk scores, survival status distributions, and heatmaps in the training set, test set, and all TCGA set.

Patients with HPV-negative OSCC were all categorized into high- or low-risk group for further analyses based on the median risk score of the training set. To evaluate the predictive capacity of the DRLs signature, survival analyses were respectively performed among three datasets: training set, testing set, and all TCGA set. Across all three datasets, the results of Kaplan–Meier curves revealed that patients in the low-risk group exhibited a better OS than patients in high-risk group (Fig. 3A–C). Except training set (*P* = 0.066, Fig. 3D), the results of PFS indicated significantly poorer outcomes in the high-risk group of testing set and all TCGA set (Fig. 3E,F). Furthermore, the risk of death of three datasets showed a similarly increased tendency in the high-risk poorer survival group than low-risk better survival group (Fig. 3G–I).

**Figure 4:**
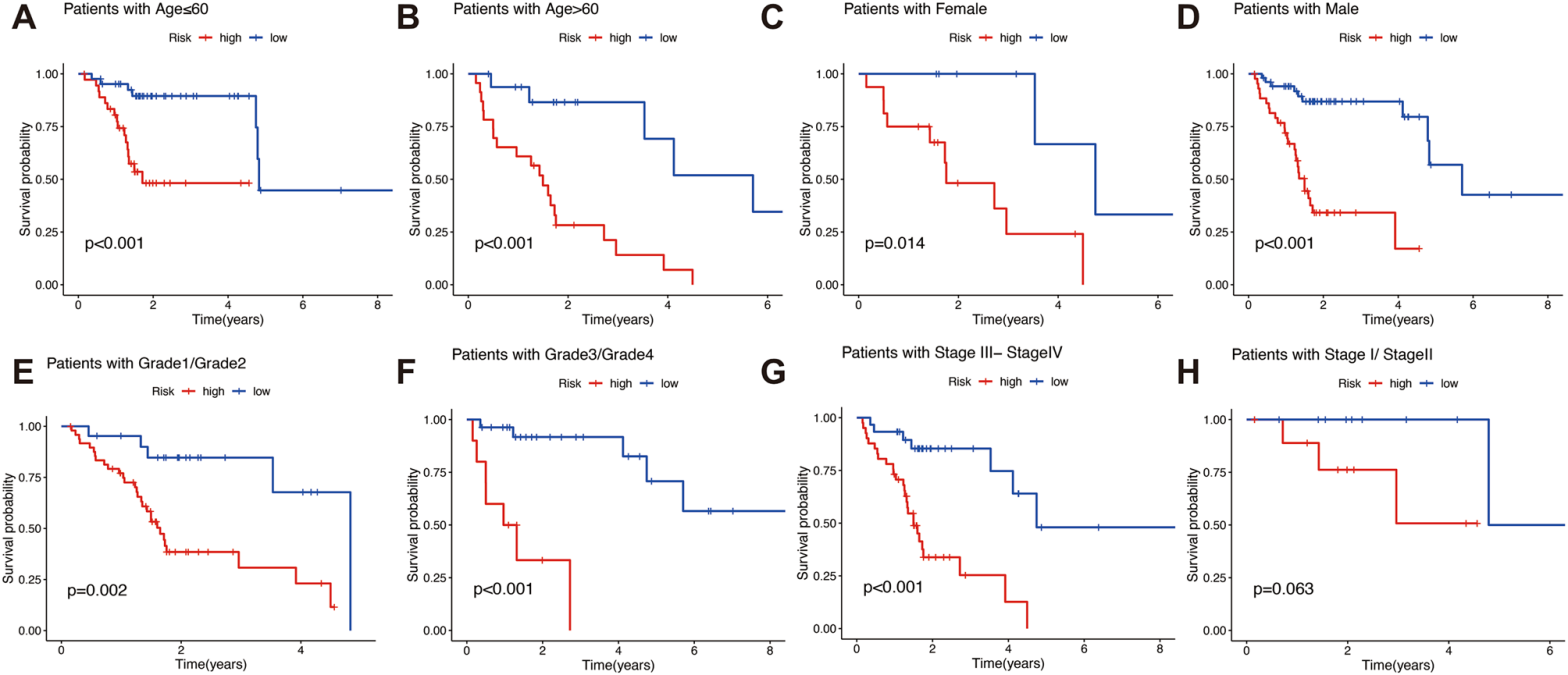
Survival analysis of subgroups stratified by different clinicopathological characteristics. (**A**, **B**) Kaplan–Meier survival curves of different age (≤ 60 years old and > 60 years old); (**C**, **D**) Kaplan–Meier survival curves of different gender (female and male); (**E**, **F**) Kaplan–Meier survival curves of different grade (grade1/grade2 and grade3/grade4); (**G**, **H**) Kaplan–Meier survival curves of different tumor stage (stageIII/stageIV and stageI/stageII).

Survival analyses were further conducted in different clinicopathological characteristics (age, gender, grade, and stage) subgroups of the DRLs signature. The results of Kaplan–Meier curves indicated that DRLs signature could effectively predicted the OS in patients with different age dimension, gender, grade, and stageIII/stageIV (Fig. 4A–F). Except for stageI/stageII patients (*P* = 0.063, Fig. 4H), there is a significantly difference between the high-risk poorer survival group and low-risk better survival group.

### Validation of DRLs signature and nomogram construction

**Figure 5:**
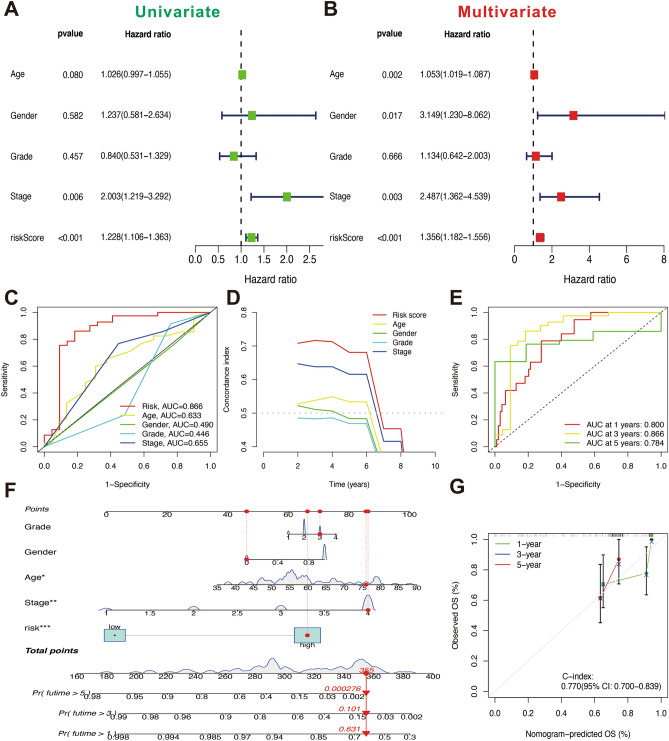
Validation of the disulfidptosis-related LncRNAs (DRLs) signature and nomogram. (**A**) Univariate Cox regression analysis for clinicopathological characteristics. (**B**) Multivariate Cox regression analysis for clinicopathological characteristics. (**C**) Time-dependent Receiver operating characteristic curve (ROC) curves of DRLs signature and clinicopathological characteristics. (**D**) The consistency index of the DRLs signature and different clinicopathological characteristics. (**E**) Area under time-dependent ROC curve (AUC) verified the prognostic accuracy of the TCGA dataset. (**F**) The constructed nomogram predicting 1-, 3-, and 5-years overall survival. (**G**) Calibration curves showed the consistency between the nomogram predicted overall survival and observed overall survival.

Univariate and multivariate COX regression analyses were performed to validate the forecasting capabilities of DRLs signature. And the results indicated that the risk score was an independent risk factor, with univariate-COX hazard ratios of 1.228(1.106–1.363) and multivariate COX hazard ratios of 1.356(1.182–1.556) (Fig. 5A,B). The time-dependent ROC curves (AUC = 0.866) indicated that the risk score was superior to all the clinicopathological characteristics in predicating OS (Fig. 5C). The consistency index of the DRLs signature was significantly higher than other clinicopathological characteristics (Fig. 5D). Time-dependent ROC curves for 1 year (AUC = 0.800), 3 years (AUC = 0.866), and 5 years (AUC = 0.784) in all TCGA dataset further indicated the outstanding predictive ability of DRLs signature (Fig. 5E). Nomogram were plotted by clinicopathological characteristics (age, grade, stage, gender) and DRLs signature (Fig. 5F). The results of 1-, 3-, and 5 years calibration plots showed a consistency between nomogram and observed OS, which demonstrated an accurate predictive performance of nomogram (95%CI 0.700–0.839, Fig. 5G).

### PCA and GSEA analysis

**Figure 6:**
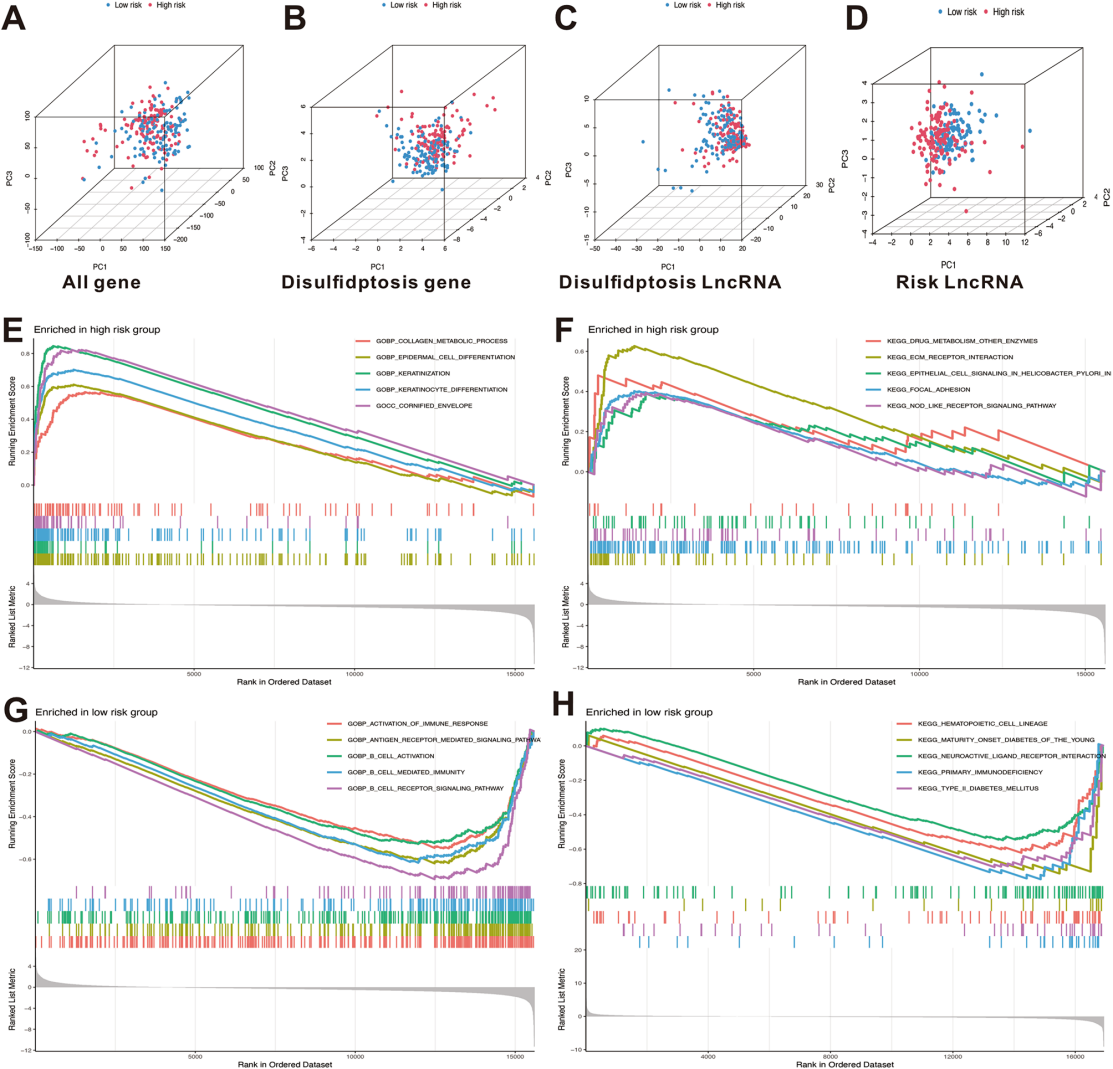
Principal component analysis (PCA) and gene set enrichment analysis (GSEA) analysis. PCA analysis of all genes (**A**), disulfidptosis-related genes (**B**), disulfidptosis-related lncRNAs (**C**), and risk lncRNAs (**D**) established the signature. GSEA analysis of the functions enrichment (**E**) and underlying signal pathways (**F**) in high-risk group. GSEA analysis of the functions enrichment (**G**) and underlying signal pathways (**H**) in low -risk group.

Four groups (all genes, disulfidptosis-related genes, disulfidptosis-related lncRNAs, and risk lncRNAs) were conducted to visualize the discriminating ability and evaluate the spatial grouping performance of DRLs signature (Fig. 6A–D). The results of PCA indicated that the DRLs signature was reliable in defining the risk thresholds compared with the other three gene sets. GSEA analysis indicated that significant enrichments related to collagen metabolic process, epidermal cell differentiation, keratinocyte differentiation, drug metabolism, epithelial cell signaling in helicobacter pylori, focal adhesion, and nod like receptor signaling pathways existed in high-risk poorer survival group (Fig. 6E,F). There was a notable enhancement of functions and pathways related to activation of immune response, antigen receptor mediated signaling pathway, B cell activation, B cell mediated immunity, and B cell receptor signaling pathway in low-risk better survival group (Fig. 6G,H).

### Immune cell infiltration and immunotherapy

**Figure 7:**
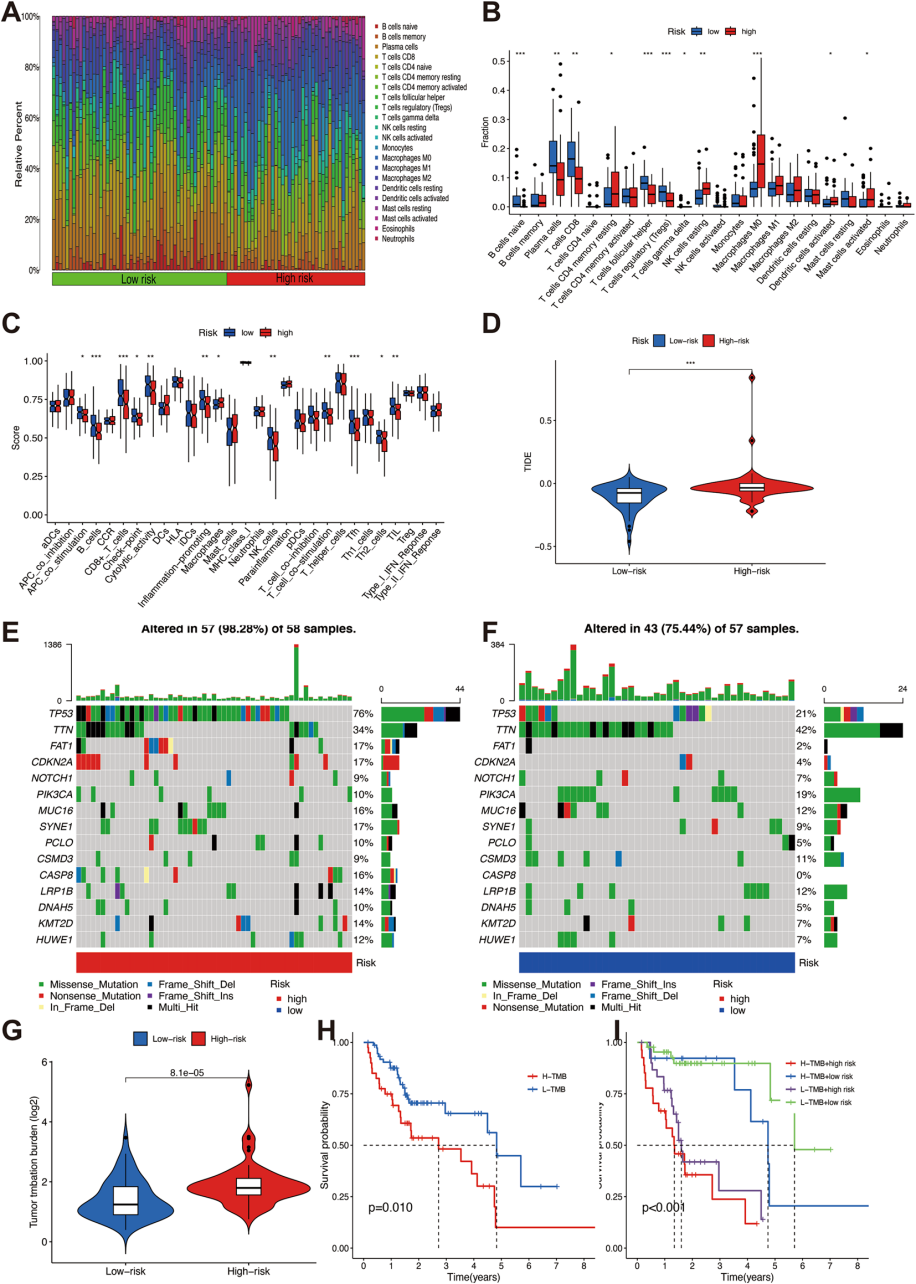
Immune landscape of the disulfidptosis-related lncRNAs signature and tumor mutational burden analysis between high- and low-risk groups. (**A**) Heatmap of the infiltrating immune cell types. (**B**) The differences scores of the infiltrating immune cells. (**C**)The comparison of immune function. (**D**) Tumor immune dysfunction and exclusion (TIDE) analysis. (**E**) Waterfall plot of top 15 mutant genes in the high-risk group. (**F**) Waterfall plot of top 15 mutant genes in the low-risk group. (**G**) Violin plots showing the difference of TMB between high- and low-risk group. (**H**) Kaplan–Meier curves of the overall survival of patients in the high-TMB and low-TMB groups. (**I**) Kaplan–Meier curves of OS combined MB and risk scores. *P < 0.05, **P < 0.01, and ***P < 0.001.

The distinctions of immune infiltration between the two risk groups of HPV-negative OSCC patients were analyzed. In terms of immune infiltrating cells. T cells CD4 memory resting, NK cells resting, Macrophages M0, Dendritic cells activated, and Mast cells activated were more abundant in the high-risk poorer survival group, while B cells naïve, Plasma cells, T cells CD8, T cells follicular helper, and T cells regulatory (Tregs) were more abundant in the low-risk better survival group(Fig. 7A,B). The results of immune functions indicated that there was higher enrichment in those immune functions (antigen-presenting cell (APC) co-stimulation, B cells, CD8^+^ T cells, Check point, iDCs, NK cells, T cell co-stimulation, follicular helper T cells (Tfh), Th2 cells, and tumor-infiltrating lymphocytes (TILs)) in low-risk better survival group compared with the high-risk poorer survival group. while inflammation − promoting immune function were significantly enriched in high-risk poorer survival group (Fig. 7C). TIDE prediction scores were negatively associated with immunotherapy, recognized as a tool for predicting the immune escape possibility. Patients with HPV-negative OSCC in the high-risk group showed a higher TIDE score than low-risk group, which indicated a poorer immunotherapy response and a higher possibility of immune escape (Fig. 7D).

### TMB analysis

TMB is critical for cancer biological activities with immense potential to serve as a predictive biomarker for immunotherapy^30^. Therefore, the gene variations of the high‐ and low‐risk group in the TCGA dataset were evaluated. The top 15 genes with the highest alteration frequency between the two risk groups were depicted in waterfall plots. The mutation rates of TP53, FAT1, and CDKN2A were obviously higher in the high-risk poorer survival group than in the low-risk better survival group, while the mutations frequency of TTN was significantly higher in the low-risk better survival group (Fig. 7E,F). Higher levels of TMB were observed in patients of high-risk poorer survival group (Fig. 7G). Survival analyses of DRLs signature revealed that the high‐TMB group had a poorer prognosis than the low‐TMB group (*P* = 0.01, Fig. 7H). The possible role of TMB played in OS were further investigated and observed that the low-TMB group combined with the low-risk group had the most beneficial survival, with 5-year OS up to 50%, whereas the high-TMB group combined with the high-risk group had the poorest outcome, with 5-year OS less than 25%. Regardless of the TMB status, the risk score was an effective prognostic factor for HPV-negative OSCC patients (*P* < 0.001, Fig. 7I).

### Drug sensitivity analysis

**Figure 8:**
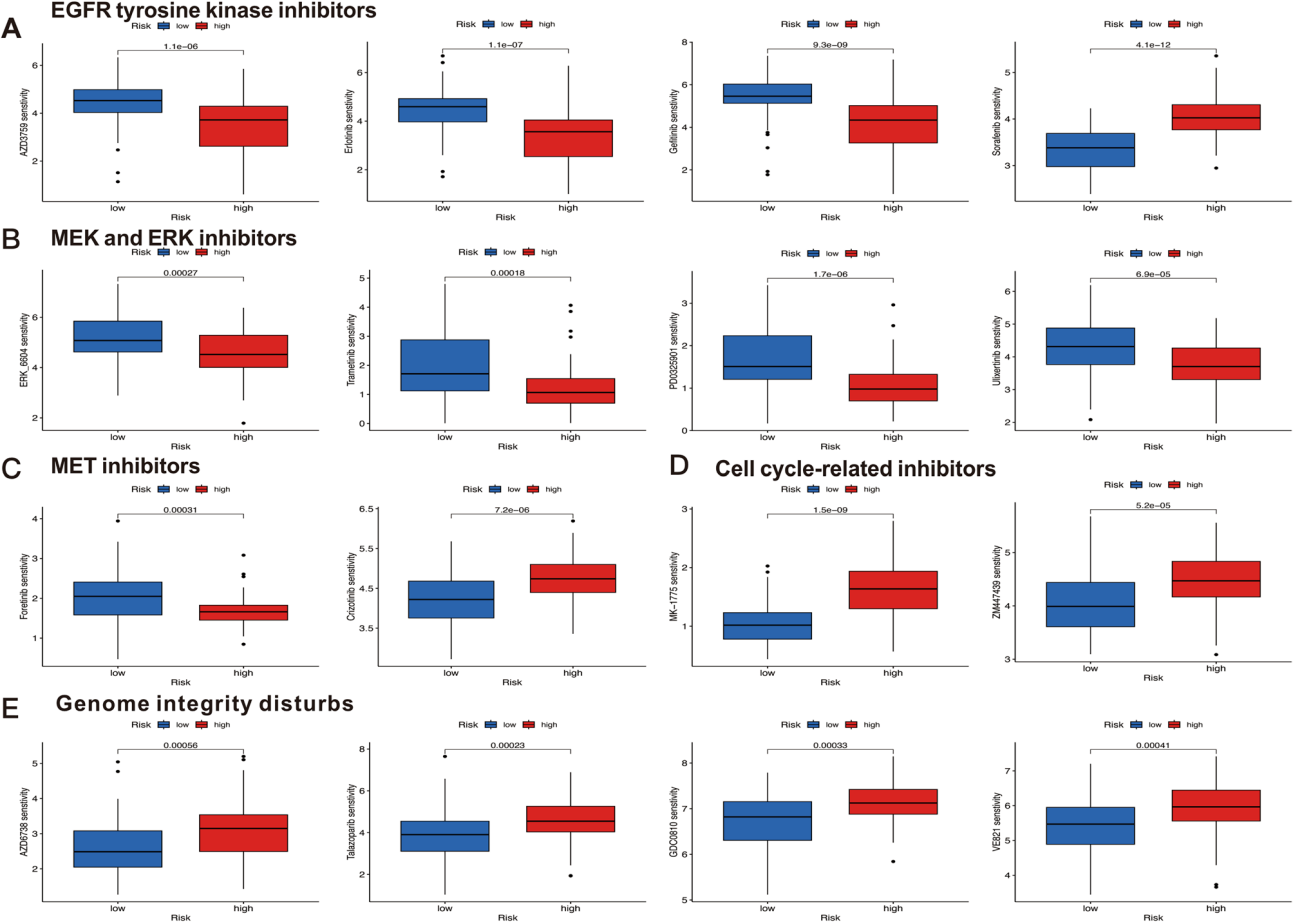
Drug sensitivities in high- and low-risk groups. (**A**) inhibitors of EGFR (**B**) inhibitors of MEK and ERK (**C**) inhibitors of MET (**D**) inhibitors of cell cycle-related kinase (**E**) disturbs of genome integrity. *P < 0.05, **P < 0.01, and ***P < 0.001.

The associations between different risk scores and their sensitivity to other anticancer drugs were investigated. Drug sensitivity scores were calculated by the calcPhenotype function based on expression profiles of patients with HPV-negative OSCC. The results indicated that patients in the high-risk poorer survival group are less sensitive to multiple sorts of chemotherapy drugs, such as EGFR inhibitors (AZD3759, Erlotinib, and Gefitinib) (Fig. 8A), MEK/ERK inhibitors (Trametinib, PD0325901, Ulixertinib, and ERK6604) (Fig. 8B) and MET inhibitors (Foretinib) (Fig. 8C). Notably, EGFR inhibitors (Sorafenib), MET inhibitors (Crizotinib), inhibitors of cell cycle-related kinases (MK-1775 and ZM447439) (Fig. 8D), and genome integrity disturbs (Talazoparib, AZD6738, VE821, and GDC0810) (Fig. 8E) are more responsive in high-risk poorer survival group compared with low-risk better survival group. These findings could facilitate developing distinct drug strategies in different risk groups based on the DRLs signature, promoting the development of precision treatment for patients with HPV-negative OSCC.

### Consensus clustering analysis of DRLs signature

**Figure 9:**
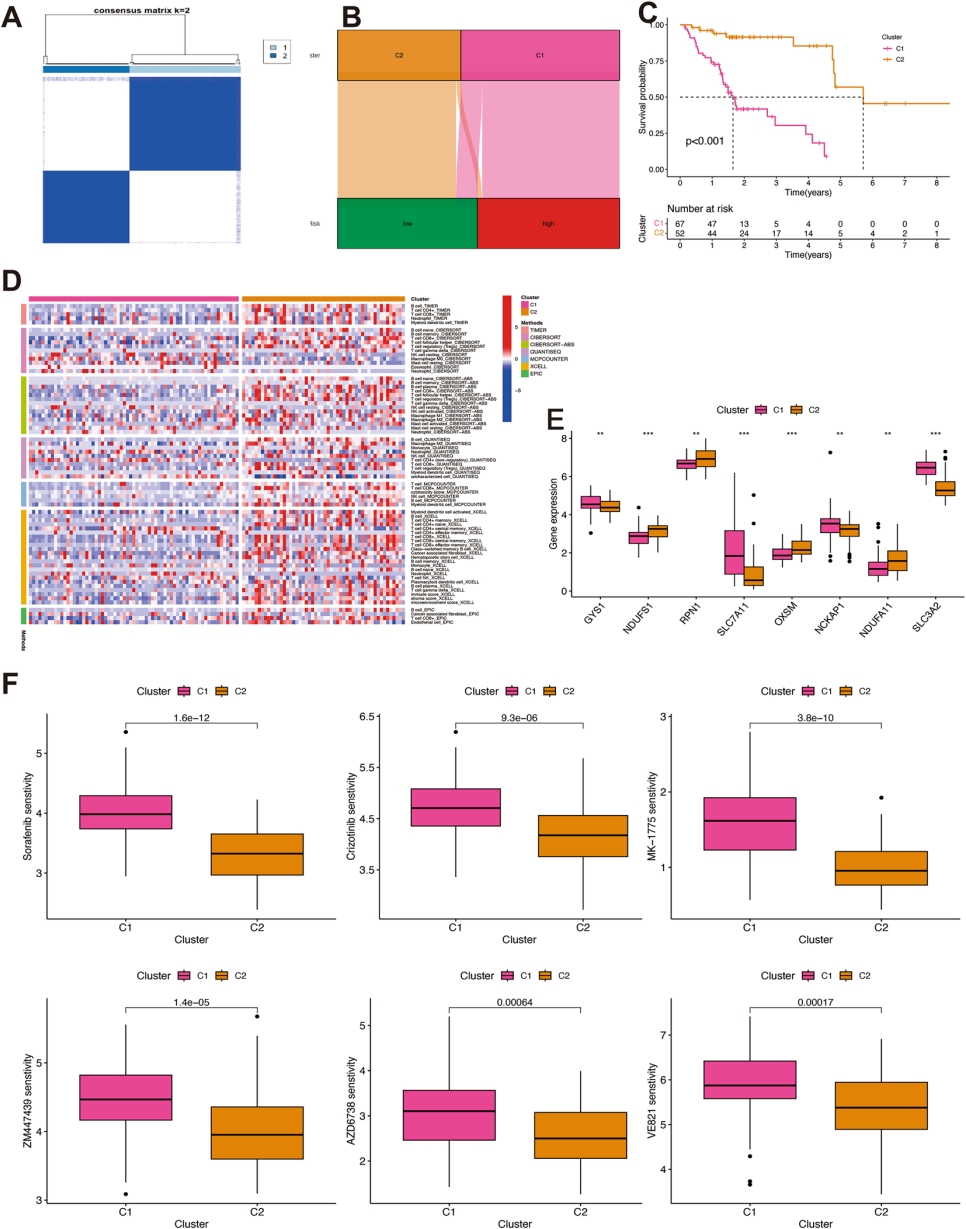
Consensus clustering analysis of disulfidptosis-related lncRNAs signature. (**A**) The consensus clustering matrix was determined as 2. (**B**) A Sankey diagram depicting the association between clusters and risk score. (**C**) The Kaplan–Meier curves for overall survival in the two clusters. (**D**) The heatmap displaying the levels of immune cells infiltration in the two clusters. (**E**) Bar plot of immune checkpoints of two clusters. (**F**) Drug sensitivity analysis of two clusters. *P < 0.05, **P < 0.01, and ***P < 0.001.

The optimal number of clusters was determined to be two (Fig. 9A). Thus, two distinct clusters among TCGA dataset were divided using a consensus clustering algorithm. Alluvial diagram was conducted to depict the relationship between two clusters and risk scores (Fig. 9B). Kaplan–Meier curves indicated that cluster 1 had a significant poorer outcome than cluster 2 (*P* < 0.01, Fig. 9C). Immune cell infiltration between the two clusters were visualized in the heatmap (Fig. 9D). The expression levels of immune checkpoint-related genes among the two clusters showed that GYS1, SLC7A11, NCKAP1, and SLC3A2 expressed higher levels in the cluster 1, while NDUFS1, RPN1, OXSM, and NDUFA11 expressed higher levels in the cluster 2 (Fig. 9E). These drugs that were more sensitive in the high-risk group (Sorafenib (EGFR inhibitors), Crizotinib (MET inhibitors), MK-1775 and ZM447439 (inhibitors of cell cycle-related kinases), and AZD6738 and VE821 (genome integrity disturbs)) were further analyzed and found to be more sensitive in cluster 1 compared with cluster 2 (Fig. 9F).

### External validation

**Figure 10:**
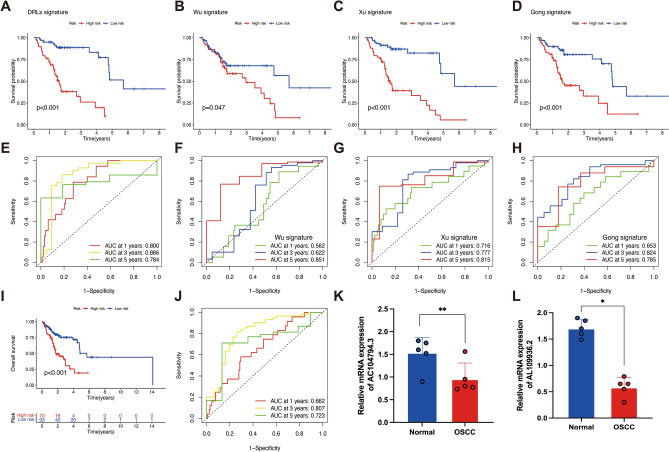
External validation of disulfidptosis-related lncRNAs (DRLs) signature and expression levels of DRLs. Kaplan–Meier survival curves of DRLs signature (**A**), Wu signature (**B**), Xu signature (**C**), and Gong signature (**D**). Time-dependent Receiver operating characteristic curve (ROC) curves of DRLs signature (**E**), Wu signature (**F**), Xu signature (**D**), and Gong signature (**H**). Kaplan–Meier survival curves of GEO dataset (**I**). ROC curves of GEO dataset (**J**). The expression level of AC104794.3 (**K**) and AL109936.2 (**L**) in adjacent normal tissues and HPV- negative OSCC tissues. *P < 0.05, **P < 0.01, and ***P < 0.001.

Three previous lncRNA signatures of OSCC were integrated for validate the practicality of DRLs signature^31–33^. Kaplan–Meier curves showed that all those signatures were significantly reliable for risk stratification (*P* < 0.05, Fig. 10A–D). Time-independent ROC analyses indicated that the DLRs signature was superior to other signatures in performance for 1- and 3- years OS assessment (Fig. 10E–H). GSE41613 dataset and GSE85446 dataset were merged as one GEO dataset to further validate the DRLs signature. Kaplan–Meier curves revealed that HPV-negative OSCC patients in the high-risk group had a poorer OS than those in the low-risk group, which is consistent with TCGA results (*P* < 0.001, Fig. 10I). The AUC values for predicting 1-, 3-, and 5-years OS were 0.662, 0.807, and 0.723, respectively (Fig. 10J). These results demonstrated that the DRLs signature had extraordinary performance in predicting OS of patients with HPV-negative OSCC.

### Validation of the expression levels of the DRLs

The expression levels of the two lncRNAs (AC104794.3 and AL109936.2) established the signature were detected by qPT-PCR. The results indicated that lncRNA AC104794.3 and lncRNA AL109936.2 were all significantly decreased in HPV-negative OSCC tissues compared with normal tissues (Fig. 10K,L).

### Experimental validation of lncRNA AC104794.3

**Figure 11:**
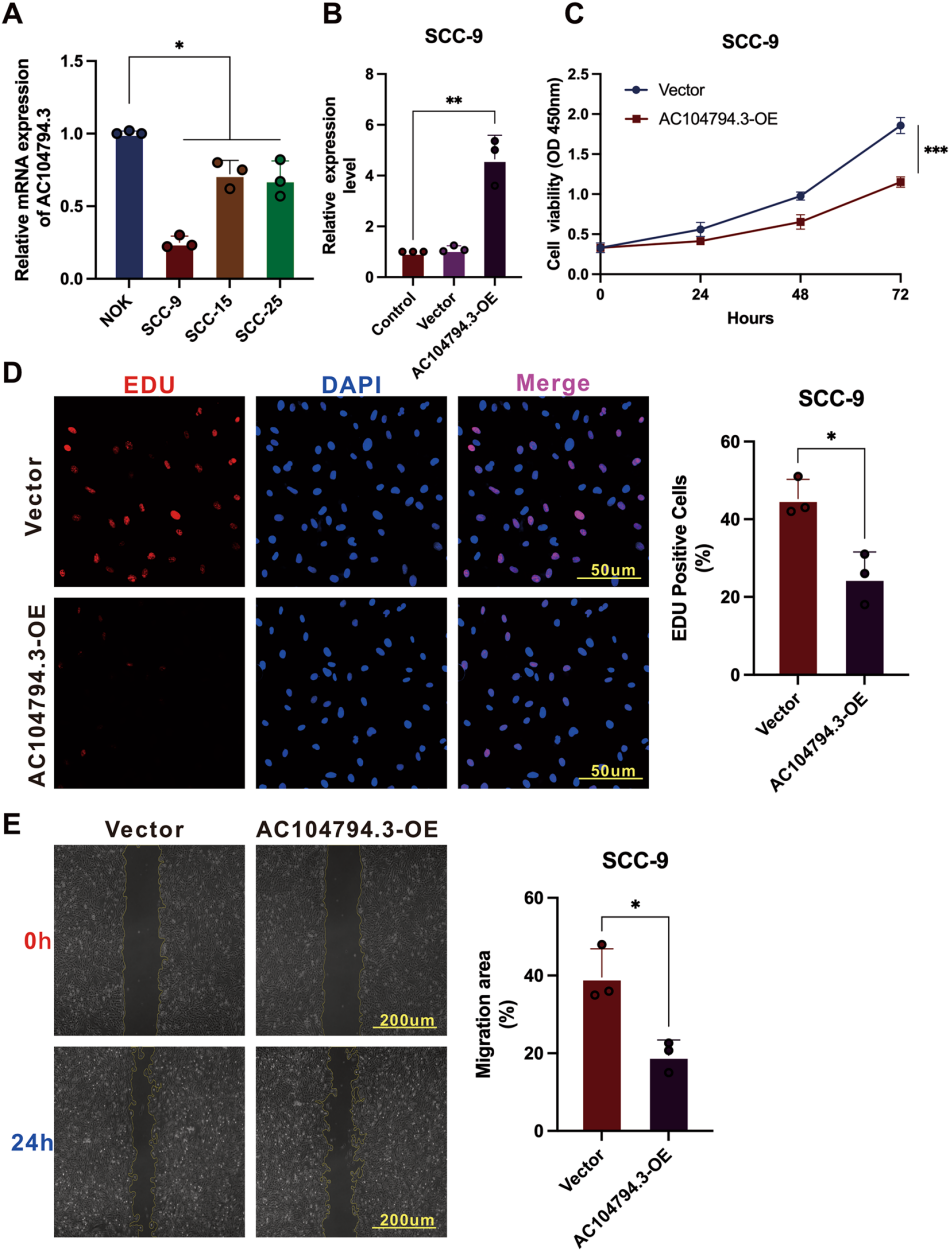
Experimental validation of lncRNA AC104794.3 in normal oral cells and OSCC cell lines. (**A**) Different lncRNA AC104794.3 expression levels in normal oral cell (NOK) and OSCC cell lines (SCC-9, SCC-15, and SCC-25). (**B**) The AC104794.3-OE expression level in SCC-9 cells after 48 h transfection. (**C**) Cell viability between vector and AC104794.3-OE groups in SCC-9 cells was determined by CCK-8 assay. (**D**) Representative images of AC104794.3-OE transfection in the cell proliferation of SCC-9 cells using EdU assays. Scale bar, 50 μm. (**E**) Representative images of migration of SCC-9 cells transfected with AC104794.3-OE. Scale bar, 200 μm. *P < 0.05, **P < 0.01, and ***P < 0.001.

The different expression levels of lncRNA AC104794.3 in NOK cells and OSCC cell lines (SCC-9, SCC-15, and SCC-25) were detected and found that lncRNA AC104794.3 was down-regulated in OSCC cell lines, especially in SCC-9 cells (Fig. 11A). To further explore the biological function of lncRNA AC104794.3 in OSCC, AC104794.3-OE were transfected into SCC-9 cells. The up-regulated expression level of lncRNA AC104794.3 were evaluated by qPT-PCR (Fig. 11B). The results of CCK-8 assays revealed that over-expression lncRNA AC104794.3 can inhibit cell viability of SCC-9 cells (Fig. 11C). Significantly lower proliferation rates were observed in lncRNA AC104794.3-OE groups than vector groups of SCC-9 cells (Fig. 11D). The effects of lncRNA AC104794.3 on the migration of OSCC were evaluated by the wound-healing assay, which indicated that lncRNA AC104794.3-OE significantly decreased the migrating area compared with vector (Fig. 11E). In conclusion, these results indicated that lncRNA AC104794.3 was down-regulated in OSCC and the expression level of lncRNA AC104794.3 might be involved in the proliferation and migration OSCC in vitro.

## Discussion

Disulfidptosis is identified as a novel type of RCD, trigged by overexpressed SLC7A11 under glucose starvation. Distinguished from other RCD, overload cystine, NADPH shortage, and sebquent disulfide stress are the prerequisites of disulfidptosis^12,34^. Disulfide stress exists not extensive in normal cellular environment. Induced by glucose starvation, SLC7A11-high cells can promote aberrant disulfide stress and consequently trigger potential disulfidptosis^35^. Although research on disulfidptosis is still in its infancy, there are compounds of studies confirmed the important between disulfidptosis and various diseases (including neurodegenerative diseases, metabolic diseases, and various cancers)^36^. Osteoclastogenesis is regulated by thioredoxin reductase 1 (TXNRD1, which mediates cystine reduction to cysteine) in osteoclast precursors. The inhibition of disulfidptosis can prohibit overactivation of osteoclasts and further prevent patients form metabolic diseases (including rheumatoid arthritis, osteoporosis, and bone metastases)^37^. High expression of SLC7A11 have been found extensively in human cancers^13^. Most of the SLC7A11-high cancers display a strong glucose dependency and are prone resistant to ferroptosis- or apoptosis-inducing therapies. However, susceptibility of these cancers to disulfidptosis illuminates new therapeutic avenues. Experimental interventions validate that inhibiting LncRNA OGFRP1 could significantly decrease disulfidptosis of cancer cells and further reduces the invasion and migration capabilities of lung cancer^38^. Preclinical findings indicate that GLUT inhibitors can regulate disulfidptosis and inhibit cancer growth^15^. BAY-876 (GLUT1 inhibitors) has been found effectively in inhibiting the growth of SLC7A11 high UMRC6 kidney cell carcinoma xenografts in mice^39^. SLC7A11-high cancer cells manifest heightened sensitivity to GLUT1 inhibitors compared to SLC7A11-low cancers. KEAP1-mutant lung cancer cells exhibit upregulated expression of SLC7A11 and are likely to accumulate disulfide stress^40^. KEAP1-mutant lung cancer is sensitive to GLUT1 inhibitors and suggests a potential therapeutic strategy to target disulfidptosis^41^. A disulfide molybdenum disulfide (MoS2) combined with a metal organic skeleton can be applied for cancer diagnostics^42^. Although researchers hypothesize that the activation of RAC1-WRC signaling pathway is essential for the progression of disulfidptosis^12^. The underlying mechanisms of disulfidptosis induce cell death require further investigation. Targeting disulfidptosis provides novel insights for cancers treatment. The translation of these findings of disulfidptosis into meaningful clinic strategy presents groundbreaking opportunities for patients. LncRNAs undertake a critical part in modulating malignant behaviors of tumors and have been deemed as possible tumor-targets in recent years^43^. DRLs signatures have been reported to forecast survival in various cancers, including colon adenocarcinoma^44^, hepatocellular carcinoma^45^, and glioma^46^. Hitherto, there are limited studies on survival significance between DRLs and HPV-negative OSCC.

In this study, 162 DRLs were identified using univariate Cox regression analysis, among them, 25 DRLs significantly related to the OS of patients with HPV-negative OSCC. LASSO analysis refined their performance and screen out 3 DRLs for multivariate Cox regression analysis. Ultimately, lncRNA AC104794.3 and lncRNA AL109936.2 were chosen to construct the signature. Literature surveys on the two DRLs were conducted from PubMed and Web of Science. AL109936.2 was recognized as a cuproptosis-related lncRNA on the previous study^47^. Few researchers reported the role played by lncRNA AC104794.3 in the onset and advancement of malignancy.

The predictive independence and precision of the DRLs signature for HPV-negative OSSC were confirmed. The results of the univariate and multivariate COX regression analysis on risk score (*P* < 0.001) and ROC curves (AUC = 0.866) revealed that DRLs signature was an independent factor and superior to other clinicopathological characteristics in predicting survival. The risk score calculated by the DRLs signature was proved more broadly applicable for providing a precise management of patient in different stratification of clinicopathological characteristics. Nomogram was created in conjunction with DRLs signature and clinicopathological characteristics to quantify the predicted OS for each patient with HPV-negative OSSC. The results of calibration curves (95%CI 0.700–0.839) indicated the reliable accuracy and specificity of DRLs signature, exhibiting that the nomogram was a robust predictive tool for HPV-negative OSSC patients.

Immune cells infiltration and the tumor microenvironment hold profound influences on the development of carcinoma. The quantity and quality of infiltrating lymphocytes are highly associated with the escape of tumor cells, the ability of immune surveillance, and progression of HPV-negative OSCC^48,49^. High-risk poorer survival group and low-risk better survival group distinguished by DRLs signature had diverse immune functions and distinct immune cell infiltration levels. Higher associations with T cells CD4 memory resting, NK cells resting, Macrophages M0, Dendritic cells activated, and Mast cells were observed in high-risk poorer survival group, whereas the B cells naïve, Plasma cells, T cells CD8, T cells follicular helper, and T cells regulatory (Tregs) were found more abundant in low-risk better survival group. Activation of T cells participated the effective antitumor immune responses based on the successful recognition of tumor antigens by T-cell receptors^50^. Humoral immunity was mediate by B cells and B cells naïve can inhibit tumor progression by secreting immunoglobulins^51^. The lower level of immune cell infiltration determined the anti-tumor immune activity and further affected the poorer survival of patients in high-risk group. Besides, most immune functions (antigen-presenting cell [APC] co-stimulation, B cells, CD8^+^ T cells, iDCs, NK cells, T cell co-stimulation, Tfh, Th2 cells, and TIL) displayed significantly higher levels in low-risk better survival group. Inflammation—promoting immune function was observed more reactive in high-risk poorer survival group. Inflammation predisposed to tumorigenesis and participated all stages of cancer progression^52^. Above findings revealed that the reduction of activated immune cell and improper immune functions activity account for the poor outcome of patients with HPV-negative OSCC. DRLs signature could effectively distinct these patients and provide feasible guidance of immunotherapy.

Only a minority of HPV-negative OSCC patients has sensitivity for immune checkpoint inhibitors. Therefore, testing whether this DRLs signature can be employed as a biomarker for immune response prediction is necessary. Higher TIDE scores were observed in high-risk group, indicating that high-risk patients are more prone to immunization escapes. Consistently, patients with high-risk are less reactive to anticancer drugs, such as most EGFR inhibitors, MEK inhibitors, and ERK inhibitors. Notably, the drugs that disturb genome integrity (Talazoparib, AZD6738, VE821, and GDC0810) are more sensitive for patients in high-risk group. These results might offer novel insights for clinical guidelines.

Progressive accumulation of somatic mutation can alter key cellular functions, resulting in tumorigenesis and development. Higher levels of TMB were observed in high-risk poorer survival group. Thus, common oncogenes with high mutation frequencies in two risk groups were further analyzed and compared. Mutation frequencies were found more abundant in the high-risk poorer survival group (98.28%) compared to low-risk better survival group (75.44%). 15 most frequently mutated genes were depicted, among them, TP53 was the top frequently mutated gene (76%) in the high-risk group compared with observed in 53% of patients in the low-risk group, which might account for the higher level of TMB in high-risk group. Mutation of TP53 gene is the most conventional genetic alterations in carcinoma. HPV-negative OSCC of older male with long-term smoking habits are easier to carry TP53 mutations^53^. The higher mutation frequencies of other genes (FAT1, CDKN2A) might be another cause of poorer outcome of patients with high-risk scores.

Subtypes of malignancy can guide preclinical and clinical therapeutic strategies and decrease treatment-related morbidity and cost^54^. Therefore, HPV-negative OSCC patients were divided into two clusters to scrutinize the similarities and dissimilarities among subtypes. Cluster 1 was observed lower infiltration of immune cells with poorer survival when compared to cluster 2. Patients in cluster 1 were sensitive to various drugs including Sorafenib, Crizotinib, MK-1775, ZM447439, AZD6738, and VE821.

Experimental validation of lncRNA AC104794.3, which constructed the risk signature in the present study were conducted. In consist with bioinformatic results, lncRNA AC104794.3 was down-regulated in OSCC cell lines (SCC-9, SCC-15, and SCC-25). For functional assays, overexpression of lncRNA AC104794.3 could inhibit the cell viability, proliferation, and migration of OSCC. These results demonstrated lncRNA AC104794.3 involved in the biological progression of OSCC, which indicated that lncRNA AC104794.3 could be a candidate biomarker and our DRLs signature may provide potential insights of targeted gene therapy for OSCC in the future.

## Conclusion

In conclusion, this study provides preliminary insights about disulfidptosis and HPV-negative OSCC. The DRLs signature can effectively predict survival, immune cell activity, and response to immunotherapy of patients with HPV-negative OSCC.

## Data Availability

The original contributions presented in the study are included in this article. Further inquiries directed to the corresponding author.
